# Expression and functions of N-type Cav2.2 and T-type Cav3.1 channels in rat vasopressin neurons under normotonic conditions

**DOI:** 10.1186/s12576-020-00775-w

**Published:** 2020-10-15

**Authors:** Kaori Sato-Numata, Tomohiro Numata, Yoichi Ueta, Yasunobu Okada

**Affiliations:** 1 Japan Society for the Promotion of Science, 5-3-1 Kojimachi, Chiyoda-ku, Tokyo, 102-0083 Japan; 2 Department of Physiology, School of Medicine, Fukuoka University, 7-45-1 Nanakuma, Jonan-ku, Fukuoka, 814-0180 Japan; 3 Department of Physiology, School of Medicine, University of Occupational and Environmental Health, 1-1, Iseigaoka, Yahatanishi-ku, Kitakyushu-shi, Fukuoka 807-8555 Japan; 4 National Institute for Physiological Sciences, 5-1 Higashiyama, Myodaiji, Okazaki, Aichi 444-8787 Japan

**Keywords:** Cav channel, Vasopressin neuron, Flufenamic acid, Action potential

## Abstract

Arginine vasopressin (AVP) neurons play essential roles in sensing the change in systemic osmolarity and regulating AVP release from their neuronal terminals to maintain the plasma osmolarity. AVP exocytosis depends on the Ca^2+^ entry via voltage-gated Ca^2+^ channels (VGCCs) in AVP neurons. In this study, suppression by siRNA-mediated knockdown and pharmacological sensitivity of VGCC currents evidenced molecular and functional expression of N-type Cav2.2 and T-type Cav3.1 in AVP neurons under normotonic conditions. Also, both the Cav2.2 and Cav3.1 currents were found to be sensitive to flufenamic acid (FFA). TTX-insensitive spontaneous action potentials were suppressed by FFA and T-type VGCC blocker Ni^2+^. However, Cav2.2-selective ω-conotoxin GVIA failed to suppress the firing activity. Taken together, it is concluded that Cav2.2 and Cav3.1 are molecularly and functionally expressed and both are sensitive to FFA in unstimulated rat AVP neurons. Also, it is suggested that Cav3.1 is primarily involved in their action potential generation.

## Background

Arginine vasopressin (AVP) neurons that are located at the supraoptic nucleus (SON) and paraventricular nucleus (PVN) in the hypothalamus of the brain are a key player in maintaining the osmolarity of body fluid in a narrow range around 300 mOsm by secreting AVP from their neuronal terminals at the posterior pituitary gland. The action potential firing involves activation of Na^+^ and Ca^2+^ conductance in the magnocellular neurosecretory cells (MNCs) consisting of AVP neurons and oxytocin (OXT) neurons [1–3]. The amount of vesicular exocytotic release of AVP from neurohypophysial terminals of MNCs is determined by the frequency and pattern of action potential firing [4] and by the entry of Ca^2+^ via voltage-gated Ca^2+^ channels (VGCCs or Cav channels) [5]. Somatodendritic expression of L-, T- and N-type VGCCs in rat supraoptic AVP neurons was suggested based on pharmacological studies on the increases in the intracellular free Ca^2+^ concentration ([Ca^2+^]_i_) in response to application of AVP [6] and pituitary adenylate cyclase-activating polypeptide (PACAP) [7]. Besides, nifedipine-sensitive L-type Ca^2+^ currents were found to be increased in rat supraoptic MNCs under dehydration conditions produced after water deprivation for 16–24 h [8]. However, it is not known which types of VGCCs are expressed and functioning during the spontaneous action potential firing in unstimulated rat AVP neurons under normotonic conditions. The present study thus aimed to address this question by cytosolic RT-PCR analysis and by whole-cell patch-clamp recordings in dissociated rat AVP neurons identified by transgenic expression of enhanced green fluorescent protein (eGFP) under the control of the AVP promoter [9]. RT-PCR, pharmacology, and gene silencing studies showed that both N-type Cav2.2 and T-type Cav3.1 Ca^2+^ channels are molecularly and functionally expressed in dissociated rat AVP neurons under normotonic conditions and also that these VGCC currents are sensitive to an anthranilic acid derivative, flufenamic acid (FFA). Furthermore, pharmacological data suggested that T-type Cav3.1 (but not N-type Cav2.2) channels are primarily involved in tetrodotoxin-insensitive spontaneous action potential firing in dissociated rat AVP neurons under normotonic conditions without any receptor stimulation.

## Methods

### Animals and preparation of acutely dissociated AVP neurons

All procedures involving animals were approved in advance by the Ethics Review Committee for Animal Experimentation of Fukuoka University and were in accordance with the guidelines of the Physiological Society of Japan. Non-transgenic female Wistar rats (Charles River Laboratories Japan, Yokohama, Japan) and heterozygous transgenic male Wistar rats, which express an AVP-enhanced green fluorescent protein (eGFP) fusion gene [9], were bred and housed under standardized conditions (12-h/12-h light/dark cycle) with food and water. For all the experiments, 4- to 5-week-old AVP-eGFP transgenic female rats were used.

Acutely dissociated AVP neurons were prepared, as described previously [10], and incubated in Ringer solution containing (in mM): 140 NaCl, 5 KCl, 1 MgCl_2_, 2 CaCl_2_, 10 HEPES and 10 glucose (adjusted to pH 7.25 with Tris, 300 mosmol kg-H_2_O^−1^, bubbled with 100% O_2_) at room temperature (22–26 °C). In all of the experiments, eGFP expression was confirmed each time under a fluorescence microscope to identify given SON neurons as AVP neurons.

### Cytosolic quantitative real-time PCR

Using the RNeasy Micro Kit (Qiagen, Tokyo, Japan), total cellular RNAs were extracted from cytosol suctioned into patch pipettes from 10 AVP neurons and pooled. RNA samples were reverse-transcribed using the ReverTra Ace qPCR RT Master Mix with gDNA Remover (TOYOBO, Osaka, Japan) according to the manufacturer’s protocols. Gene-specific primers used for PCR were designed with Primer3 software (https://bioinfo.ut.ee/primer3/) and NCBI BLAST (https://blast.ncbi.nlm.nih.gov/Blast.cgi) to identify complementary sequences in the rat genome. The following primers were used: 5′-CAGTGCGTGTTTGTTGCTATCCG-3′ (forward)/5′-TTTGGGGATGTAACACCTCAGCG-3′ (reverse) for *Cav1.1* (GenBank accession no. NM_053873.1, product size 558); 5′-CAGCTGTTTGGTGGAAAGTTCA-3′ (forward)/5′-TGTTGATCTTGGTAGTGGGTGG-3′ (reverse) for *Cav1.2* (GenBank accession no. NM_012517.2, product size 457); 5′-TCAATGGAAGCGTGTGTCCTCG-3′ (forward)/5′-ACATTCTGTCTTCTGGGGCTGG-3′ (reverse) for *Cav1.3* (GenBank accession no. NM_017298.1, product size 403); 5′-CGAGGATTTCGGTGTCTCTACCCA-3′ (forward)/5′-TTGCAGACACTGGATGGTGAAGG-3′ (reverse) for *Cav1.4* (GenBank accession no. NM_053701.1, product size 404); 5′-CAAGAACTCCGGGATCCAAAAAC-3′ (forward)/5′-CAGCTCCACCCTTTGCGATTT-3′ (reverse) for *Cav2.1* (GenBank accession no NM_012918.3, product size 428); 5′-AGGCCAGACATGAAGACACACA-3′ (forward)/5′-TTGCCTTCCTTGCTTGAGTCCT-3′ (reverse) for *Cav2.2* (GenBank accession no. NM_001195199.1 and NM_147141.1, product size 422); and 5′-TGCTGTATAACGGCATCCGCTC-3′ (forward)/5′-TCTGGTTGTCCAGGACGCTAGT-3′ (reverse) for *Cav2.3* (GenBank accession no NM_019294.2, product size 570); 5′-ACGGGACCTGAAGAAGTGCTAC-3′ (forward)/5′-ATCGACTCTCCGGAAGTTCTGC-3′ (reverse) for *Cav3.1* (GenBank accession no. NM_001308302.1 and NM_031601.4, product size 586); and 5′-ATCAATCCCACCATCATCCGCA-3′ (forward)/5′-ACCTTGGCTTTCCTGTGCTGTA-3′ (reverse) for *Cav3.2* (GenBank accession no NM_153814.2, product size 579); and 5′-CAGCCTACAGACCACACTGGAA-3′ (forward)/5′-TCTTCCTTTTGCTCGCAGCATC-3′ (reverse) for *Cav3.3* (GenBank accession no NM_020084.3, product size 587). As a positive control, we amplified the glyceraldehyde-3-phosphate-dehydrogenase (GAPDH) sequence with the following set of primers: 5′-CATGCCGCCTGGAGAAACCTGCCA-3′ (forward)/5′-GGGCTCCCCAGGCCCCTCCTGT-3′ (reverse) (GenBank accession no. NM_017008.3, product size 429). As a negative control, we performed RT-PCR without reverse transcriptase. PCR was performed with 0.02 U μL^−1^ of KOD -Plus- Neo *Taq* (TOYOBO). Amplification was carried out in a thermal cycler (Tadvanced 96 SG: Biometra, Göttingen, Germany) under the following conditions: initial heating at 94 °C for 2.5 min, followed by 40 cycles of denaturation at 94 °C for 15 s, annealing at 60 °C for 30 s and extension at 68 °C for 1 min, and then final extension at 68 °C for 5 min. The products of RT-PCR were electrophoresed on a 2% agarose gel and then cloned into the pGEM-T Easy vector (Promega, Tokyo, Japan) after purification with the Wizard SV Gel and PCR Clean-Up System (Promega). Plasmids were purified with the Wizard Plus Minipreps DNA Purification System (Promega) and used as templates for sequencing by FASMAC (Kanagawa, Japan). Quantitative real-time PCR and data analysis were performed using an ABI 7500 Fast Real-Time PCR System (Applied Biosystems, Foster City, CA, USA). Amplifications were performed using KOD SYBR qPCR Mix (TOYOBO) according to the manufacturer’s instructions of annealing at 57 °C in a 20-μL reaction volume containing 100 ng cDNA. Gene-specific primers were same as those used in the PCR.

### Modification of gene expression and culture of acutely dissociated AVP neurons

To reduce the expression of rat *Cav2.2* and *Cav3.1*, siRNA-mediated knockdown was performed in AVP neurons. Fifteen minutes after acutely dissociated AVP neurons were plated on coverglasses, Ringer solution were replaced with Neurobasal Plus medium (Gibco, NY, USA) supplemented with 0.5 mM l-glutamine, 25 μM glutamate, 1/50-diluted B-27 Plus Supplement (50X; Gibco), 100 U mL^−1^ penicillin, and 0.1 mg mL^−1^ streptomycin (Gibco). For siRNA transfection, Lipofectamine RNAiMAX transfection reagent (Invitrogen, CA, USA) was employed according to the manufacture’s protocols. siRNAs conjugated with DY547 for *Cav2.2* (Cat No. J-097736-10), *Cav3.1* (Cat No. L-089308-02), and negative control (Cat No. D-001810-01) were designed and synthesized by Thermo Fischer Scientific (Tokyo, Japan). AVP neurons after siRNA transfection were incubated in a 95% air/5% CO_2_ atmosphere at 37 °C for 24–60 h, and were used for experiments.

### Electrophysiology

The patch electrodes had a resistance of around 1–3 MΩ. Currents or voltages were recorded using an Axopatch 200B amplifier (Axon Instruments, CA, USA) coupled to DigiData 1440A A/D and D/A converters (Axon Instruments). Currents or voltage signals were filtered at 5 kHz and digitized at 20 kHz. pClamp software (version 9.0.2: Axon Instruments) was used for command pulse control, data acquisition and analysis. For the measurements of Ca^2+^ channel currents, whole-cell voltage-clamp recordings were performed at room temperature. The time course of current activation was monitored by repetitively applying (every 10 s) alternating pulses (0.3-s duration) of − 10 mV or + 10 mV from a holding potential of − 80 mV. To observe voltage dependence of the current profile, step pulses were applied (every 10 s) from the holding potential of − 80 mV to test potentials of − 60 or − 70 mV to + 70 mV for 0.3 s in 10 mV increments. Series resistance (< 10 MΩ) was compensated (to 70–80%) to minimize voltage errors. The intracellular (pipette) solution contained (in mM): 160 NMDG-phosphate, 4 MgCl_2_, 40 HEPES, 10 EGTA, 12 phosphocreatine, 0.1 leupeptin, 2 ATP-Tris salt and 0.4 GTP-Tris salt (280 mosmol kg-H_2_O^−1^, adjusted to pH 7.2 with phosphoric acid). Normotonic extracellular (bath) solution (300 mosmol kg-H_2_O^−1^) contained (in mM): 60 or 156 TEA-Cl, 10 HEPES, 50 or 2 BaCl_2_, 10 glucose and 80 or 0 mannitol (adjusted to pH 7.3 with TEA-OH). For the measurements of membrane potential and spontaneous firing, perforated whole-cell current-clamp recordings were performed at 32–35 °C controlled by using a thermo-controller (WARNER Instruments, Hamden, CT) with the pipette solution containing (in mM) 99 K_2_SO_4_, 31 KCl, 5 MgCl_2_, 0.2 EGTA, and 5 HEPES (280 mosmol kg-H_2_O^−1^, adjusted to pH 7.4 with KOH). Pipette solution was placed in the tip of the pipette by capillary action (~ 5 s), and then pipettes were backfilled with nystatin-containing (200 μg mL^−1^) pipette solution. The normotonic bath (300 mosmol kg-H_2_O^−1^) solution contained (in mM) 120 NaCl, 5 KCl, 2 CaCl_2_, 1 MgCl_2_, 10 glucose, 10 HEPES, and 100 mannitol (adjusted to pH 7.3 with NaOH).

### Statistical analysis

Data were given as the mean ± SEM of observations (*n*). Statistical differences of the data were evaluated using one-way ANOVA followed by a Bonferroni-type post hoc multiple comparisons and considered to be significant at *P* < 0.05.

### Chemicals

All chemicals were prepared on the day of the experiments by diluting stock solutions with 1000-fold concentrations. The stocks of flufenamic acid (Sigma-Aldrich, Tokyo, Japan), TTA-P2 (Alomone labs, Jerusalem, Israel), and ML218 (Sigma-Aldrich) were made in dimethyl sulfoxide (DMSO: Wako Pure Chemical, Osaka, Japan) and kept at − 20 °C. The stocks of tetrodotoxin (Sigma-Aldrich), ω-conotoxin GVIA (PEPTIDE INSTITUTE, INC., Osaka, Japan), ω-agatoxin-IVA (PEPTIDE INSTITUTE, INC.), ω-conotoxin MVIIC (PEPTIDE INSTITUTE, INC.), and SNX-482 (PEPTIDE INSTITUTE, INC.) were made in distilled water and kept at − 20 °C. Ni^2+^ (Wako Pure Chemical) was directly dissolved in the solution for each experiment and used within that day.

## Results

### N-type Cav2.2 and T-type Cav3.1 channels are molecularly and functionally expressed in rat AVP neurons under unstimulated normotonic conditions

**Fig. 1:**
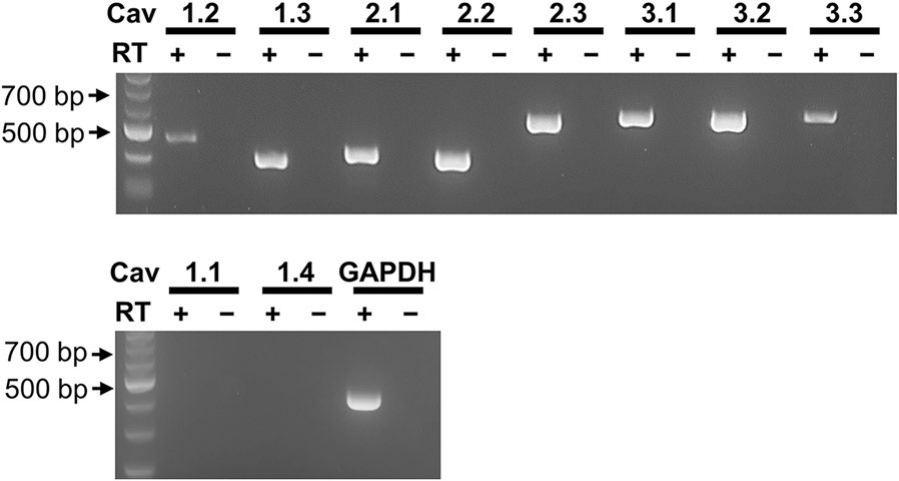
Expression of mRNAs encoding VGCCs in AVP neurons exposed to normotonic solution. The pooled cytosol of 10 AVP neurons was used in RT-PCR for *Cav1.1* (558 base pairs (bp)), *Cav1.2* (457 bp), *Cav1.3* (403 bp), *Cav1.4* (404 bp), *Cav2.1* (428 bp), *Cav2.2* (422 bp), *Cav2.3* (570 bp), *Cav3.1* (586 bp), *Cav3.2* (579 bp), *Cav3.3* (587 bp), or *GAPDH* (429 bp). *RT *(+) and *RT *(*−*) represent lanes with and without reverse transcriptase (*n* = 5). Data are representative of octuplicate experiments

First, we performed RT-PCR for VGCC genes on the total RNA extract from pooled cytosols suctioned into patch pipettes from 10 individual eGFP-expressing AVP neurons isolated from the transgenic rat. As shown in Fig. 1, we actually observed bands of the size predicted for the mRNAs encoding all the brain members of VGCC: *Cav1.2*, *Cav1.3*, *Cav2.1*, *Cav2.2*, *Cav2.3*, *Cav3.1*, *Cav3.2* and *Cav3.3*, whereas no PCR product was amplified when reverse transcriptase was omitted from the reactions. In contrast, PCR products for the skeletal muscle-type *Cav1.1* and retinal-type *Cav1.4* were not detected. The above eight Cav channel genes expressed in AVP neurons were completely matched by sequence analysis to the sequences corresponding to their respective rat Cav channels. These data indicate that unstimulated rat AVP neurons exhibit molecular expression of L-type Cav1.2 and Cav1.3, P/Q-type Cav2.1, N-type Cav2.2, R-type Cav2.3, and T-type Cav3.1, Cav3.2 and Cav3.3 channels.

**Fig. 2:**
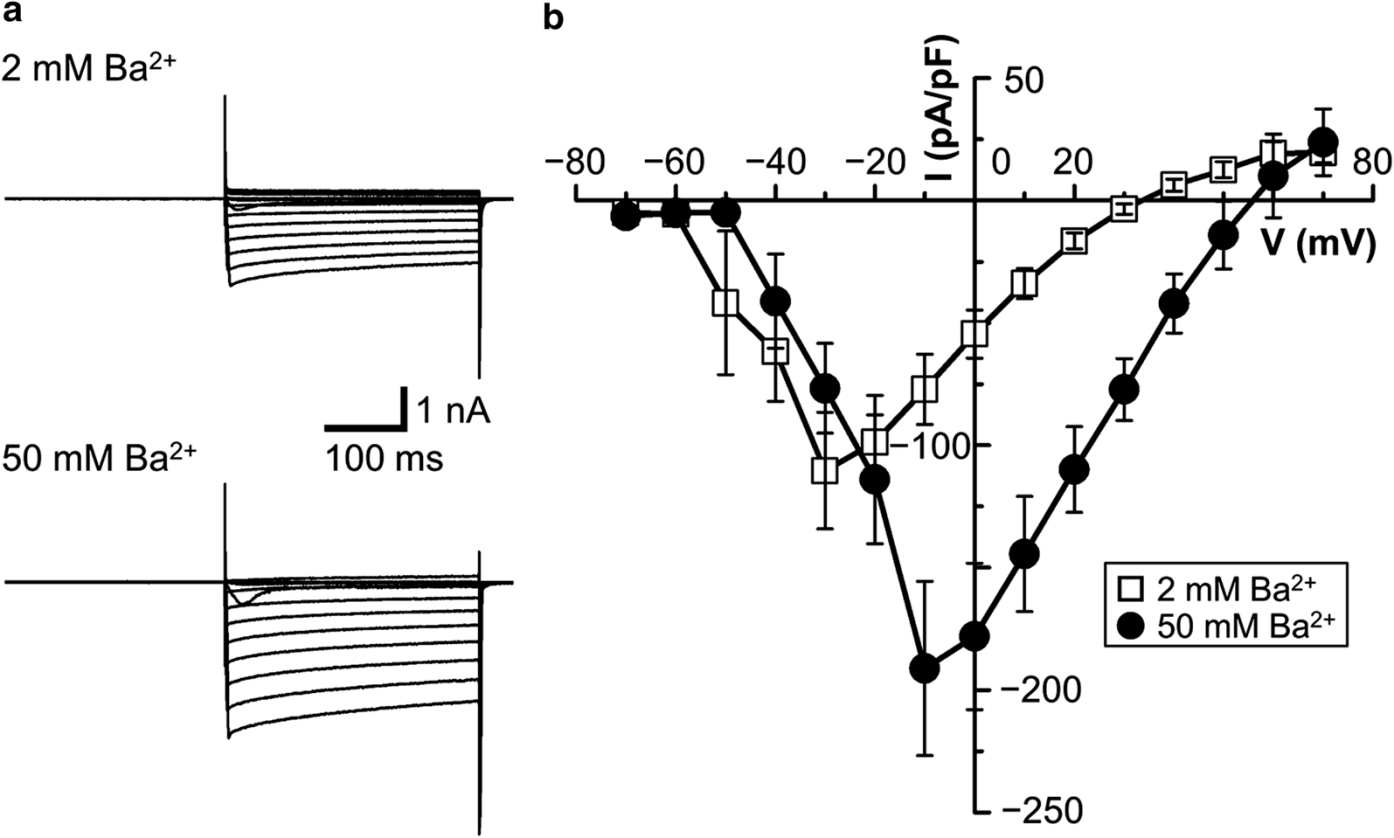
VGCC currents in AVP neurons exposed to normotonic solution.** a** Representative traces of current responses to step pulses from *−* 60 to + 70 mV in the presence of 2 mM (upper panel) and 50 mM (lower panel) Ba^2+^. **b** Current–voltage relationships for peak VGCC currents recorded with 2 mM Ba^2+^ (*n* = 7) and 50 mM Ba^2+^ (*n* = 8)

**Fig. 3:**
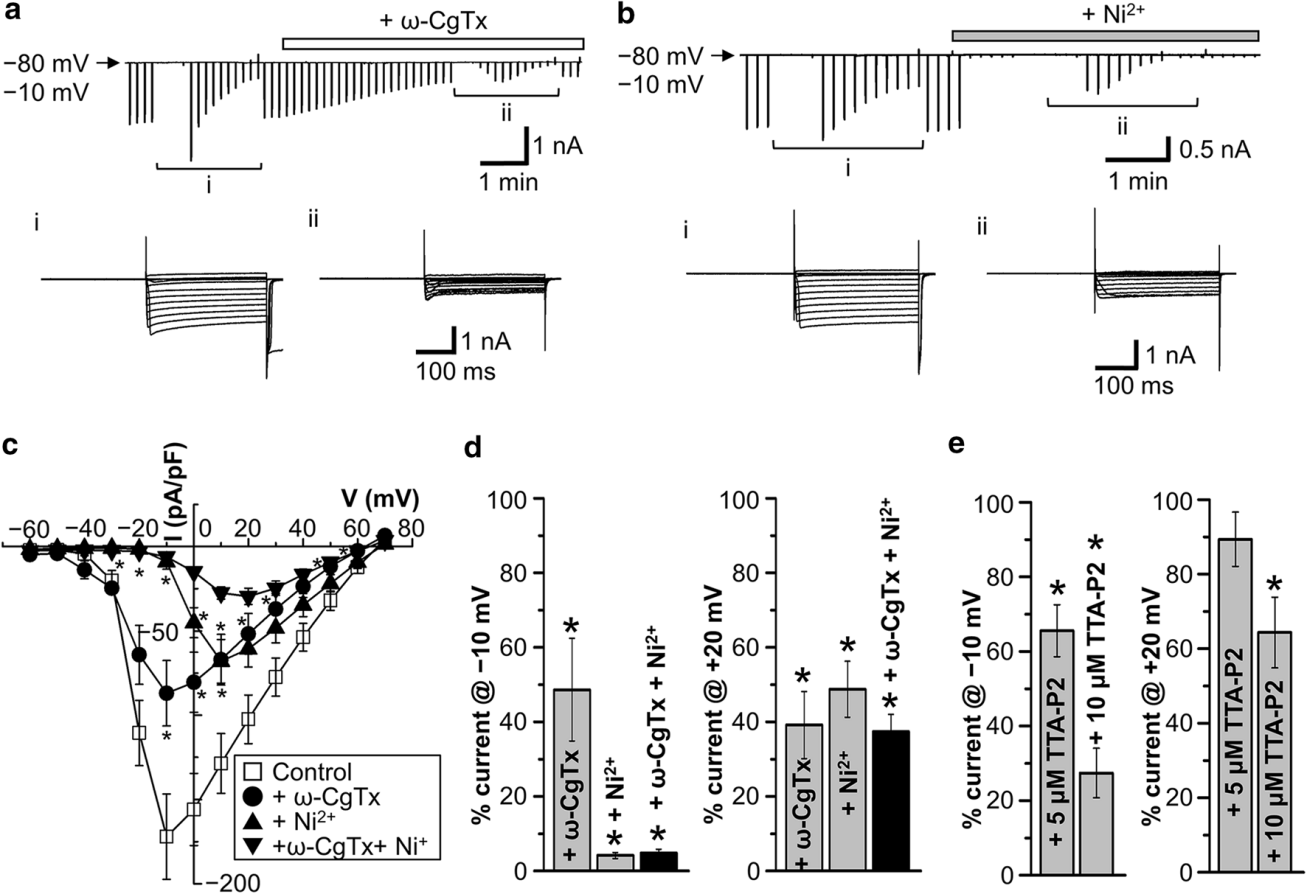
Effects of ω-CgTx, Ni^2+^ or ω-CgTx *plus* Ni^2+^ on VGCC currents in unstimulated AVP neurons. **a, b** Representative records of currents before and after application of N-type Ca^2+^ channel blocker ω-conotoxin GVIA (ω-CgTx 0.5 μM: **a**) or T-type Ca^2+^ channel blocker Ni^2+^ (3 mM: **b**) to AVP neurons exposed to normotonic solution containing 50 mM Ba^2+^. Inset panels given below the current traces in **a** and **b** represent expanded current responses to step pulses from – 60 to + 70 mV applied at *i* and *ii*. **c** Current–voltage relationships for peak VGCC currents (*n* = 6–12). *P < 0.05 vs. *Control*. **d** Percent currents at − 10 mV (left panel) and + 20 mV (right panel) in the presence of ω-CgTx (*n* = 6), Ni^2+^ (*n* = 8), and ω-CgTx plus Ni^2+^ (*n* = 7) compared to the control currents (*Control*) in the absent of these blockers. *P < 0.05 vs. *Control*. **e** Percent currents at − 10 mV (left panel) and + 20 mV (right panel) in the presence of 5 and 10 μM TTA-P2 (*n* = 5) compared to the control currents (*Control*) in the absent of this blocker. *P < 0.05 vs. *Control*

Next, we observed depolarization-induced Ba^2+^ currents to examine functional expression of VGCCs by whole-cell patch-clamp recordings in dissociated rat AVP neurons exposed to extracellular solution containing Ba^2+^ in place of Ca^2+^. As shown in Fig. 2, in the presence of 2 mM and 50 mM Ba^2+^, voltage-gated Ba^2+^ currents became activated at > − 60 mV and > − 50 mV, and the peak currents were observed at around − 30 mV and − 10 mV, respectively. Since the currents with 50 mM Ba^2+^ were much larger than those with 2 mM Ba^2+^, hereafter, whole-cell VGCC currents were observed in the presence of 50 mM Ba^2+^. As shown in Fig. 3, VGCC currents were partially but significantly suppressed by either application of ω-conotoxin GVIA (ω-CgTx, 0.5 μM: a, c), which is a specific blocker of N-type Ca^2+^ channels [11–13], or that of Ni^2+^ (3 mM: b, c), which is known to exhibit an inhibitory effect on T-type Ca^2+^ channels [14, 15], and mostly suppressed by simultaneous applications of both ω-CgTx and Ni^2+^ (c). As shown in Fig. 3c, it is noted that the peak VGCC currents were observed at − 10 mV in the absence of any blockers (*Control*) and presence of ω-CgTx (+ *ω-CgTx*), but those were shifted to + 10 mV and + 20 mV in the presence of Ni^2+^ and of Ni^2+^ *plus* ω-CgTx (+ *ω-CgTx* + *Ni*^*2*+^), respectively. Also, the threshold voltage for VGCC activation was − 50 to − 40 mV for *Control* and + *ω-CgTx*, whereas that was positively shifted to − 30 to − 20 mV for + *Ni*^*2*+^ and + *ω-CgTx* + *Ni*^*2*+^. The inactivation time constant for ω-CgTx-sensitive currents (36.0 ± 4.7 ms, *n* = 7) was faster than that of Ni^2+^-sensitive currents (70.0 ± 10.4 ms, *n* = 8). These results indicate that the VGCC currents are composed of Ni^2+^-sensitive low-voltage-activated and ω-CgTx-sensitive high-voltage-activated ones. Consistently, as summarized in Fig. 3d, the VGCC currents recorded at − 10 mV were almost abolished by Ni^2+^ but only partially inhibited by ω-CgTx, whereas those recorded at + 20 mV were less effectively suppressed by Ni^2+^. As seen in Fig. 3 (c, d), there remains a minor part of high-voltage-activated VGCC currents resistant to Ni^2+^ and ω-CgTx, presumably L-, P/Q- and/or R-type ones. In addition, as summarized in Fig. 3e, application of 5 μM 3,5-dichloro-*N*-[1-(2,2-dimethyl-tetrahydro-pyran-4-ylmethyl)-4-fluoro-piperidin-4-ylmethyl]-benzamide (TTA-P2), which is known as a T-type-selective blocker [16], significantly, though partially, suppressed VGCC currents recorded at − 10 mV (left panel) without significantly affecting the currents recorded at + 20 mV (right panel). The VGCC currents recorded at − 10 mV were markedly inhibited by 10 μM TTA-P2 (Fig. 3e: left panel), but those recorded at + 20 mV were also mildly suppressed (right panel). These results are consistent with the previous observations in dorsal root ganglion cells that TTA-P2 selectively inhibited T-type Ca^2+^ currents at < 10 μM, but TTA-P2 at ≥ 10 μM inhibited, though less markedly, high-voltage-activated VGCC currents as well [17]. These pharmacological observations suggest that the VGCC currents are predominantly, though not all, composed of N-type (Cav2.2) and T-type Ca^2+^ channel activities in AVP neurons under normotonic conditions.

**Fig. 4:**
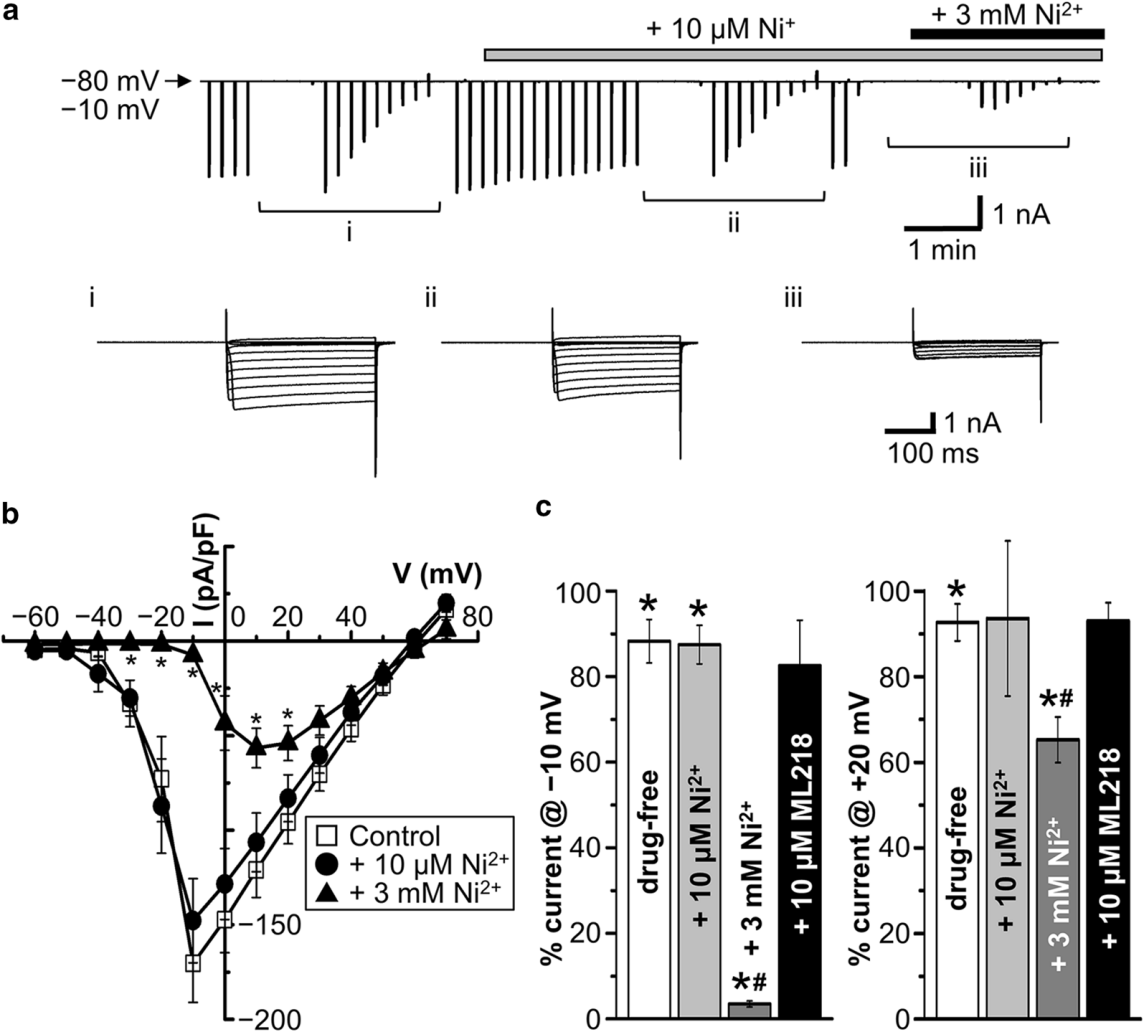
Effects of Ni^2+^ and ML218 on VGCC currents in unstimulated AVP neurons. **a** Representative record of currents before and after application of a low concentration (10 μM) or a high concentration (3 mM) of Ni^2+^ to AVP neurons exposed to normotonic solution containing 50 mM Ba^2+^. Inset panels given below the current trace represent expanded current responses to step pulses from *−* 60 to + 70 mV applied at *i, ii* and *iii*. **b** Current–voltage relationships for peak VGCC currents (*n* = 8–12). *P < 0.05 vs. *Control*. **c** Percent currents at − 10 mV (left panel) and + 20 mV (right panel) after perfusion of control solutions (*n* = 8) containing no blockers (*drug-free*: *n* = 8), 10 μM Ni^2+^ (*n* = 12), 3 mM Ni^2+^ (*n* = 8), and 10 μM ML218 (*n* = 9) compared to the control currents (*Control*) recorded before perfusion. *P < 0.05 vs. *Control*. ^#^*P* < 0.05 vs. *drug-free*

Subsequently, we examined which of the T-type VGCCs of Cav3.1, Cav3.2 and Cav3.3 is functionally expressed in AVP neurons by observing the effects of 10 μM and 3 mM Ni^2+^ (Fig. 4a,b). The VGCC currents showed a weak rundown by 11.8 ± 5.1% at − 10 mV and 7.32 ± 4.3% at + 20 mV (Fig. 4c: *drug-free*) after perfusion of bath solution over several minutes. Peak VGCC currents recorded at − 10 mV were not significantly affected by the application of 10 μM Ni^2+^ (Fig. 4a–c), at which the Cav3.2 channel is known to be specifically blocked [14]. However, the currents recorded at − 10 mV almost abolished and those recorded at + 20 mV were less effectively but significantly suppressed by application of 3 mM Ni^2+^ (Fig. 4b,c), at which all Cav3.1, Cav3.2 and Cav3.3 channels are known to be inhibited [14, 15]. Although 3 mM Ni^2+^ may not be precisely specific to T-type Ca^2+^ channels, Ni^2+^ almost completely suppressed the peak VGCC current observed at − 10 mV at this concentration. Taken together with the TTA-P2 effects (Fig. 3e), it appears that the Ni^2+^-sensitive component observed at − 10 mV represents mainly T-type Ca^2+^ currents. In contrast to Ni^2+^ and TTA-P2, ML218 (10 μM), which is a known blocker of both Cav3.2 and Cav3.3 channels [18], failed to significantly affect the depolarization-induced Ba^2+^ currents (Fig. 4c), suggesting that only Cav3.1 is functioning as T-type Ca^2+^ channels in AVP neurons under normotonic conditions.

**Fig. 5:**
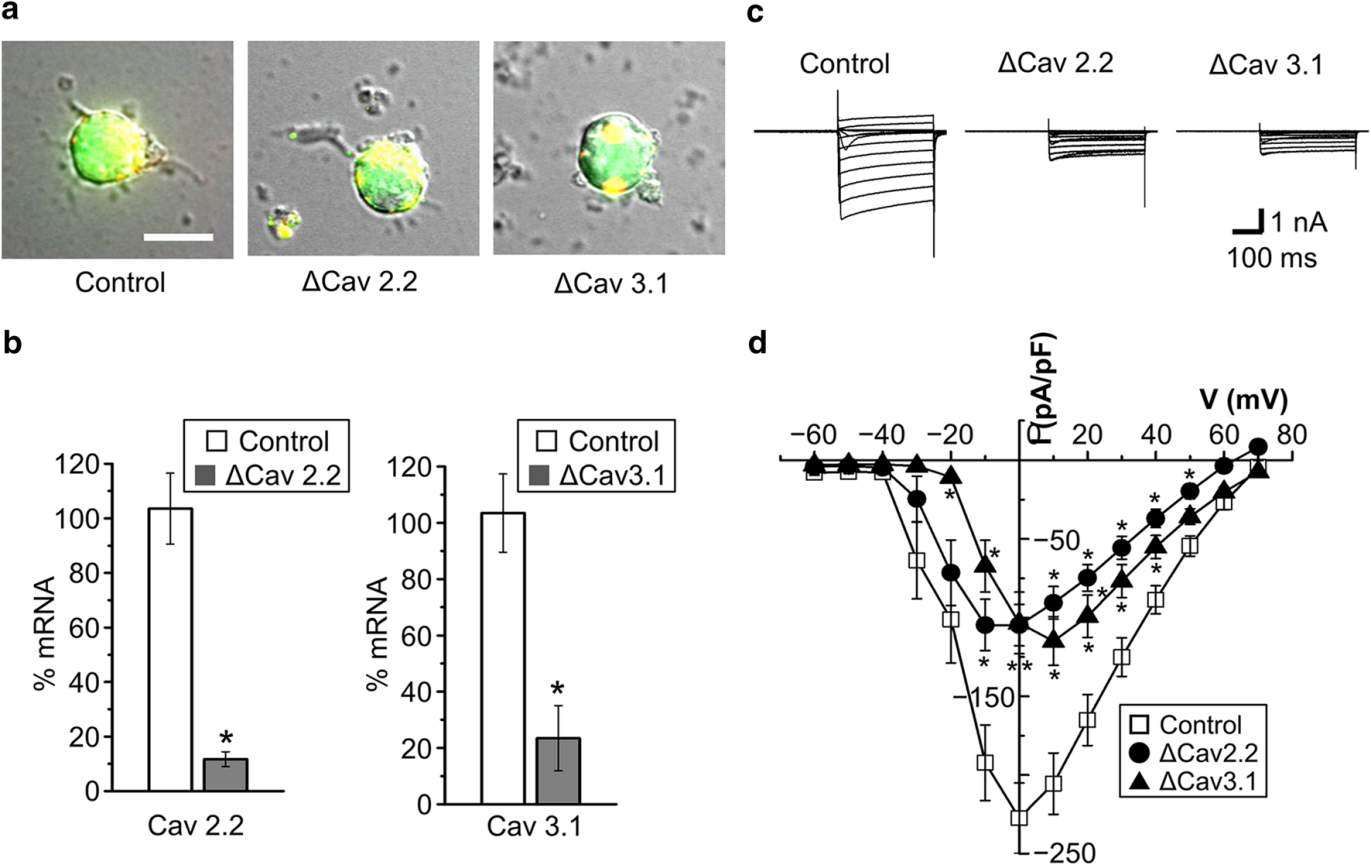
Effects of siRNA-mediated knockdown of *Cav2.2* or *Cav3.1* on VGCC currents in unstimulated AVP neurons. **a** Representative fluorescence phase-contrast images among GFP-expressing AVP neurons transfected with DY547-labeled siRNAs for negative control (*Control*: left), *Cav2.2* (*ΔCav2.2*: middle), and *Cav3.1* (*ΔCav3.1*: right). We selected yellowish AVP neurons expressing GFP (green) and DY547 (red) together. Scale bar in the left panel indicates 10 μm. **b** Quantitative real-time PCR analysis of the expression of *Cav2.2* and *Cav3.1* in AVP neurons transfected with siRNAs specific to *Cav2.2* (*ΔCav2.2*: left panel) and *Cav3.1* (*ΔCav3.1*: right panel) as well as in negative control siRNA-transfected (*Control*) AVP neurons. Rat GAPDH mRNA was used as an internal control (*n* = 5–6). *P < 0.05 vs. *Control*. **c** Representative traces of current responses to step pulses from – 60 to + 70 mV in AVP neurons transfected with siRNAs specific to *Cav2.2* (*ΔCav2.2*: middle panel) and *Cav3.1* (*ΔCav3.1*: right panel) as well as in negative control siRNA-transfected (*Control*: left panel) AVP neurons in normotonic solution containing 50 mM Ba^2+^. **d** Current–voltage relationships for peak VGCC currents in AVP neurons transfected with siRNAs for *Cav2.2* (*ΔCav2.2*) and *Cav3.1* (*ΔCav3.1*) as well as with negative control siRNA (*Control*) (*n* = 8–19). *P < 0.05 vs. *Control*

We then examined the effects of siRNA-mediated single knockdown of *Cav2.2* and *Cav3.1* in AVP neurons. To do so, we selected yellowish neurons, as shown in Fig. 5a, expressing GFP-tagged AVP (in green) and DY547-tagged siRNAs (in red) for negative control (*Control*: left panel), *Cav2.2* (middle panel), and *Cav3.1* (right panel). Knockdown efficacy was confirmed by real-time PCR for Cav2.2 mRNAs (Fig. 5b, left panel) as well as for Cav3.1 mRNAs (Fig. 5b, right panel). As shown in Fig. 5 (c, d), VGCC currents were markedly suppressed by siRNA-mediated knockdown for *Cav2.2* (*ΔCav2.2*) or *Cav3.1* (*ΔCav3.1*). The peak currents for *ΔCav2.2* were observed at − 10 to 0 mV, but those for *ΔCav3.1* were shifted to + 10 mV (Fig. 5d). On balance, it is concluded that both high-voltage-activated N-type Cav2.2 and low-voltage-activated T-type Cav3.1 channels are not only molecularly, but also functionally expressed in rat AVP neurons under normotonic conditions.

### Both Cav2.2 and Cav3.1 channels are sensitive to FFA in rat AVP neurons

**Fig. 6:**
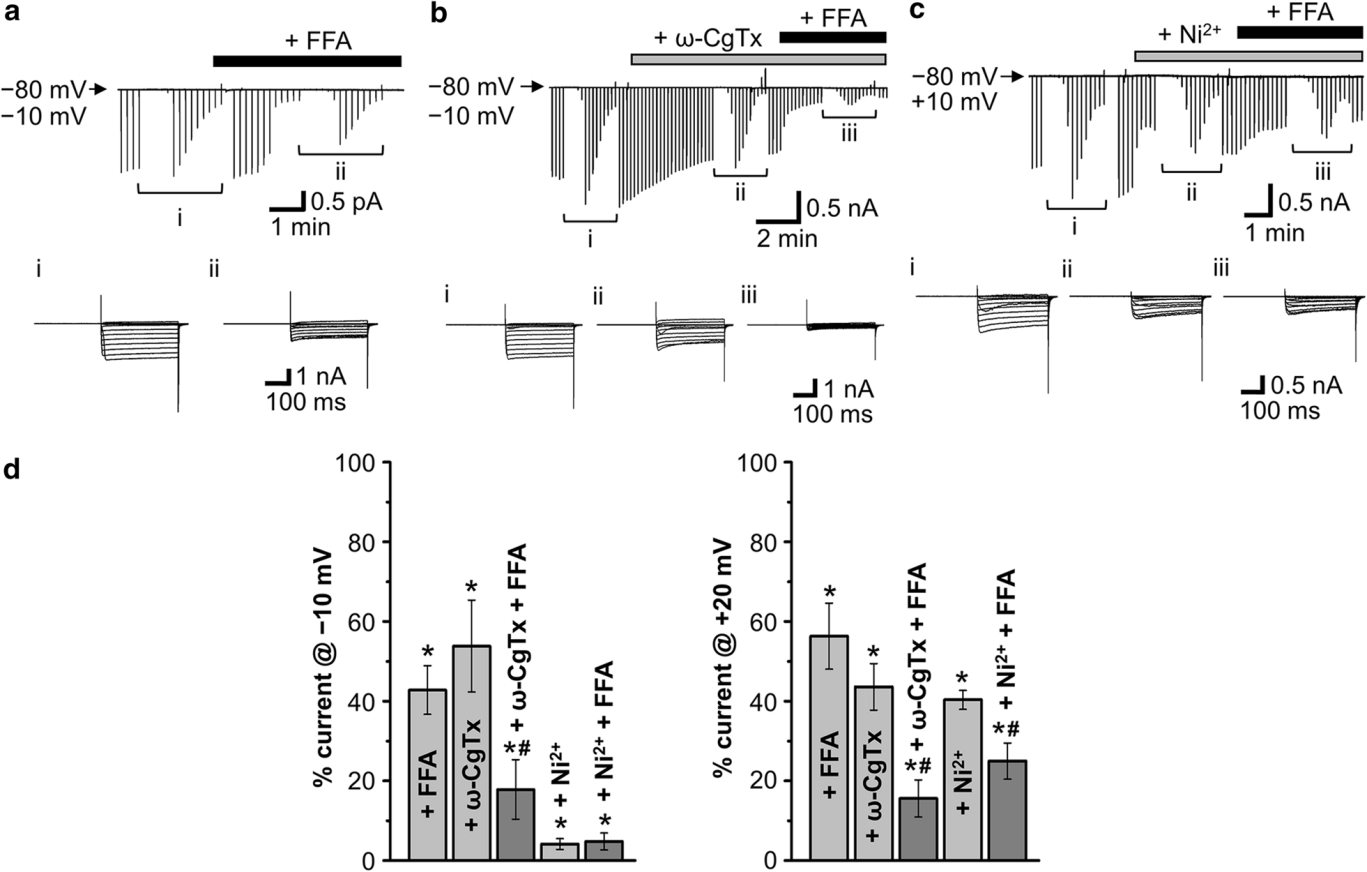
Effects of flufenamic acid (FFA) on VGCC currents in unstimulated AVP neurons. **a** Representative record of currents before and after application of 100 μM FFA (upper panel) in AVP neurons exposed to normotonic solution containing 50 mM Ba^2+^. Inset panels given below the current trace represent expanded current responses to step pulses from – 60 to + 70 mV applied at *i* and *ii*. **b, c** Representative records of currents before and after application of FFA in the absence and presence of 0.5 μM ω-CgTx (**b**) or 3 mM Ni^2+^ (**c**). Inset panels given below the current traces represent expanded traces of current responses to step pulses from – 60 to + 70 mV applied at *i*, *ii* and *iii*. **d** Percent currents at − 10 mV (left panel) and + 20 mV (right panel) in the presence of FFA (*n* = 9), ω-CgTx (*n* = 11), ω-CgTx *plus* FFA (*n* = 11), Ni^2+^ (*n* = 8), and Ni^2+^ *plus* FFA (*n* = 8) compared to the control currents (*Control*) in the absence of these blockers. *P < 0.05 vs. *Control*. ^#^*P* < 0.05 vs. ω-CgTx alone or Ni^2+^ alone

We next examined effects of FFA, which was reported to inhibit not only a number of non-selective TRP cation channels in a variety of cell types [19–21], but also L-type Ca^2+^ channels in smooth muscle cells [22], on VGCC currents in rat supraoptic AVP neurons under conditions where extracellular and intracellular cations are replaced with TEA and NMDG, respectively, and thus non-selective cation channel currents were minimized. As shown in Fig. 6(a, d), to our surprise, FFA (100 μM) partially but significantly suppressed VGCC currents in dissociated AVP neurons under normotonic conditions. The VGCC currents were also found to be significantly suppressed by 70 μM FFA by 59.6 ± 7.7% at − 10 mV and 42.3 ± 8.0% at + 20 mV (*n* = 5). At 100 μM, FFA markedly inhibited the ω-CgTx-insensitive component of VGCC currents (Fig. 6b) but less markedly the Ni^2+^-insensitive component (Fig. 6c). As summarized in Fig. 6d, FFA significantly suppressed both the ω-CgTx-insensitive components recorded at + 20 mV and − 10 mV, whereas FFA inhibited the Ni^2+^-insensitive component recorded at + 20 mV but not that recorded at − 10 mV. Taken together, it is clear that the FFA-sensitive component of currents are predominantly composed of T-type and N-type VGCC currents. These results show that both N-type Cav2.2 and T-type Cav3.1 Ca^2+^ channels functionally expressed in the unstimulated rat AVP neurons are sensitive to FFA.

### FFA-sensitive T-type Cav3.1 channels are involved in spontaneous firing activity in unstimulated AVP neurons

**Fig. 7:**
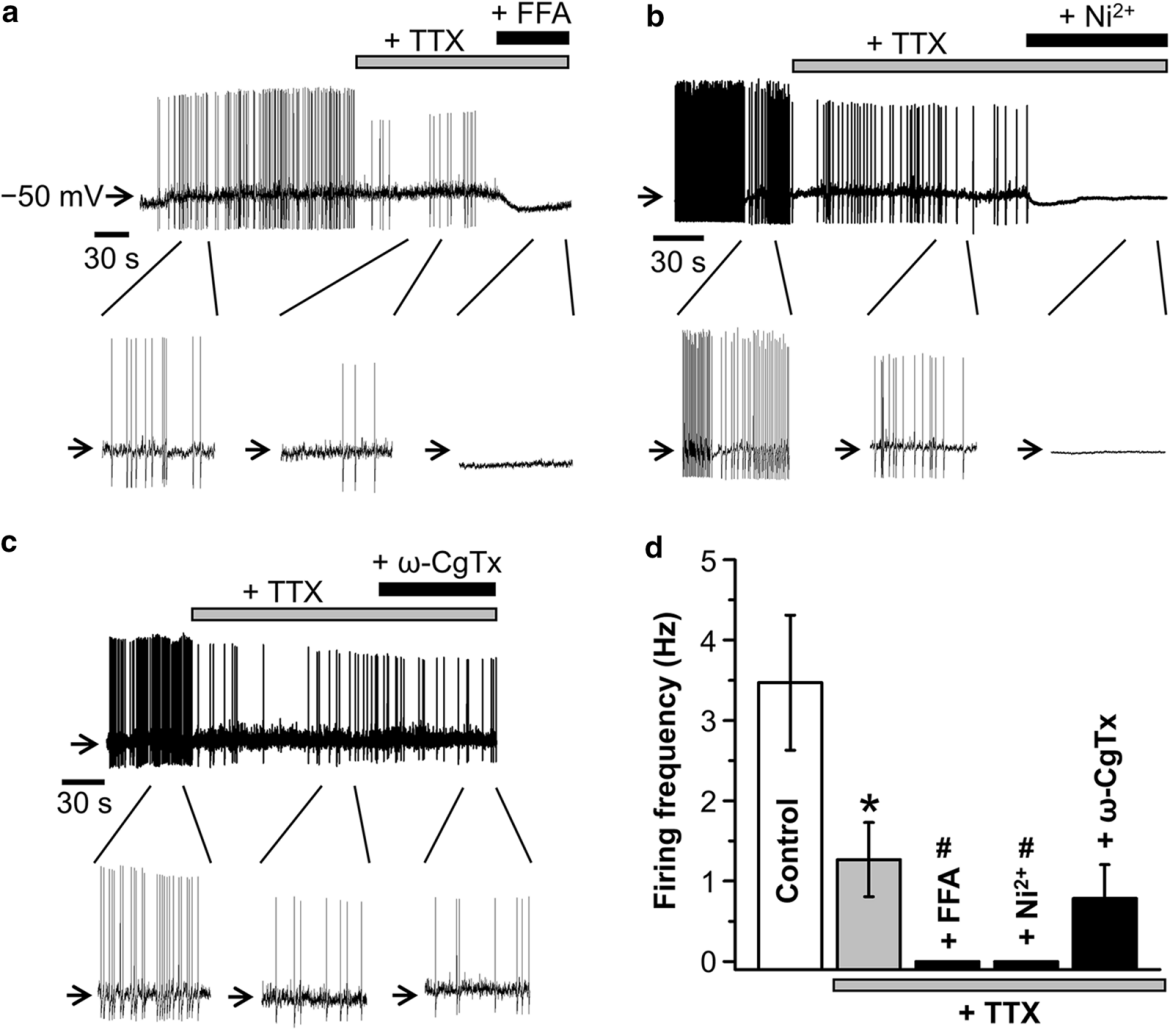
Effects of FFA on the TTX-insensitive component of action potential firing in unstimulated AVP neurons. **a–c** Representative records of spontaneous action potential firing before and after application of 0.5 μM tetrodotoxin (TTX) and then of 100 μM FFA (**a**), 3 mM Ni^2+^ (**b**), or 0.5 μM ω-CgTx (**c**) in the presence of TTX in AVP neurons exposed to normotonic solution containing 2 mM Ca^2+^. Inset panels given below the traces of membrane potential changes represent expanded traces for the indicated parts of the records. **d** The averages of firing frequency (Hz) in the absence (*Control*) and presence of TTX (+ *TTX*) or TTX *plus* FFA (+ *FFA*), TTX *plus* ω-CgTx (+ *ω-CgTx*), or TTX *plus* Ni^2+^ (+ *Ni*^*2*+^). *P < 0.05 vs. *Control* (*n* = 7–24). ^#^*P* < 0.05 vs. TTX alone

To examine whether these FFA-sensitive VGCCs are essentially involved in the spontaneous action potential firing in unstimulated AVP neurons, we observed the effects of FFA on the spontaneous firing in dissociated rat AVP neurons by nystatin-perforated whole-cell current-clamp recordings under normotonic conditions. As shown in Fig. 7, the spontaneous firing was found to be very prominent under normotonic conditions. The spontaneous firing was clearly inhibited by a voltage-gated Na^+^ channel blocker, tetrodotoxin (TTX, 0.5 μM). However, even in the presence of TTX, firing activity was maintained, though it became prominently less frequent. This TTX-insensitive firing activity was almost completely eliminated by additional application of FFA (100 μM: Fig. 7a) or Ni^2+^ (3 mM: Fig. 7b), but not affected by ω-CgTx (0.5 μM: Fig. 7c). As summarized in Fig. 7d, the firing frequency was significantly reduced by TTX and virtually nullified by FFA or Ni^2+^ added on top of TTX. These results indicate that not only TTX-sensitive Na^+^ channels, but also FFA- and Ni^2+^-sensitive Cav3.1 channels are involved in spontaneous firing activity in dissociated AVP neurons under unstimulated normotonic conditions.

## Discussion

Release of AVP and oxytocin (OXT) from MNCs in the hypothalamus is evoked by Ca^2+^ influx through VGCCs [5, 23, 24]. Electrophysiological studies on VGCC currents showed that the soma of rat supraoptic MNCs functionally express T-, N-, L-, P/Q- and R-type Ca^2+^ channels [25–29]. However, increasing evidence has shown that there are some differences in important properties including ion channel activities and firing patterns between AVP and OXT neurons [30]. Thus, further studies have been warranted to be performed on VGCC activities in AVP neurons by distinguishing from those in OXT neurons, and vice versa. In both rat supraoptic AVP and OXT neurons, molecular expression of mRNAs for non-T-type, high-voltage-activated Ca^2+^ channels were first reported by Glasgow et al. (1999) [31]. On the other hand, functional expression of T-type Ca^2+^ channels was observed in guinea pig supraoptic neurons, which likely represent AVP neurons displaying a depolarizing potential fired phasically [32]. Expression of T-, N- and L-type Ca^2+^ channels in rat supraoptic AVP neurons was indirectly suggested by observing sensitivity to VGCC blockers of the increases in the intracellular Ca^2+^ concentration ([Ca^2+^]_i_) in response to AVP [6] and PACAP [7]. Direct electrophysiological studies showed functional expression of L-, N- and P/Q-type of Ca^2+^ channels in the neurohypophysial nerve terminals isolated from rats under unstimulated normotonic conditions [5]. However, these studies have not directly addressed the expression of VGCCs in AVP neurons largely devoid of their nerve terminals. In the present study, for the first time, expression of VGCCs was examined under unstimulated normotonic conditions in dissociated rat AVP neurons that were distinctly identified by transgenic eGFP expression. RT-PCR analysis showed that mRNAs for L-, P/Q-, N-, R- and T-type Ca^2+^ channels (*Cav1.2* and *Cav1.3*, *Cav2.1*, *Cav2.2*, *Cav2.3*, and *Cav3.1* to *Cav3.3*, respectively) were detected in the cytosol (Fig. 1). In dissociated rat AVP neurons, whole-cell VGCC currents were significantly inhibited, in an additive manner (Fig. 3), by ω-CgTx, which is a known blocker specific for N-type Cav2.2 channels [11–13], and by a high concentration (3 mM) of Ni^2+^, which is known to predominantly block T-type Cav3.1, Cav3.2 and Cav3.3 channels [14, 15]. The VGCC currents sensitive to ω-CgTx and to Ni^2+^ exhibited rapid and moderate inactivation rates (with *τ* of 36 and 70 ms), which match the inactivation properties of T- and N-type Ca^2+^ channels, respectively [33]. Expression of T-type Ca^2+^ channels in unstimulated rat AVP neurons is consistent with that of Ni^2+^-sensitive Ca^2+^ channels observed in unstimulated guinea pig AVP-like magnocellular neurons [32]. In contrast, Ni^2+^ failed to suppress the VGCC currents at 10 μM (Fig. 4), the concentration of which was reported to block Cav3.2, but not Cav3.1 and Cav3.3, channels [14]. Also, the VGCC currents were insensitive to ML218 (Fig. 4c), which blocks Cav3.2 and Cav3.3 channels [18]. Taken together, it is concluded that Cav2.2 and Cav3.1 channels are predominantly functioning in the plasma membrane of unstimulated rat AVP neurons under normotonic conditions.

Low-voltage-activated or low-threshold T-type Ca^2+^ channels are activated at lower voltages than high-threshold L-, P/Q-, N- and R-type Ca^2+^ channels. Under physiological recording conditions, the apparent activation threshold for T-type Ca^2+^ channels is − 50 to − 70 mV (see Reviews [33, 34]). However, when the extracellular Ba^2+^ concentration was increased from 1 and 2 mM to 10 and 40 mM, the threshold activation voltage for T-type Ca^2+^ channels was shown to be shifted by around 10 and 20 mV, respectively, to a positive direction, because of the effect of such high concentrations of Ba^2+^ on surface charge screening [35, 36]. In agreement with these facts, in the present study, the Ba^2+^ currents exhibited a threshold activation voltage of > − 60 and > − 50 mV in the presence of 2 and 50 mM Ba^2+^, respectively (Fig. 2). As a consequence, the voltage at which the peak currents were observed was also shifted from around − 30 mV to − 10 mV, when extracellular Ba^2+^, was increased from 2 to 50 mM (Fig. 2), suggesting that such a high concentration of Ba^2+^ caused formation of a positive surface potential, thereby shifted the voltage actually subjected to the Ca^2+^ channel protein toward a more negative one in the present study as well.

The present study also, for the first time, demonstrated that both N-type Cav2.2 and T-type Cav3.1 Ca^2+^ channels in AVP neurons are sensitive to FFA (Fig. 6). FFA has long been used therapeutically as one of the top prescribed non-steroidal anti-inflammatory drugs (NSAIDs) which exhibit anti-inflammatory, analgesic and antipyretic effects [37]. When 200 mg FFA was orally administered to young healthy persons, the peak plasma concentration was reported to reach 6 to 20 μg mL^−1^, or 21 to 71 μM, within 1.5 h [38]. Since FFA was shown to largely suppress both T- and N-type VGCC currents at 70 and 100 μM in the present study in vitro, it is feasible that the endogenous VGCC activities, especially T-type one, in the axon terminal in situ are sometimes partially suppressed by the plasma FFA after oral administration, because the posterior pituitary region exists outside the blood–brain barrier [39].

Somatodendritic action potentials of rat supraoptic MCNs were previously shown to arise from co-activation of Na^+^ and Ca^2+^ conductances [2]. However, it has not been known whether this is the case for AVP neurons distinguished from OXT neurons. In the present study, it was shown that the spontaneous firing in rat AVP neurons under unstimulated normotonic conditions is caused by the activities both of TTX-sensitive Na^+^ channels and of FFA- and Ni^2+^-sensitive T-type Cav3.1 Ca^2+^ channels (Fig. 7). The time interval between spikes of around 490 ms may be sufficient to attain ≥ 90% recovery of T-type Ca^2+^ channel activity from inactivation, in light of previous data of around 90% recovery from short inactivation at 400 ms after firing of neuronal T-type Ca^2+^ channels [40]. When FFA was applied to AVP neurons in the presence of TTX, the membrane became hyperpolarized (Fig. 7a), whereas the resting potential was not much affected by Ni^2+^ and ω-CgTx (Fig. 7b, c). Since FFA is known to suppress a number of the cation-permeable TRP channel family [19–21], it is suggested that FFA-induced hyperpolarization was caused by its inhibitory action to some of TRP cation channels in AVP neurons. Further studies are required to elucidate the contribution of each type of VGCC to the firing activity under hypertonic or prolonged dehydration/hydration conditions.

## Conclusions

In dissociated rat AVP neurons under normotonic conditions, N-type Cav2.2 and T-type Cav3.1 VGCCs were found to be expressed and predominantly functioning, and be sensitive to FFA. Also, it is suggested that T-type Cav3.1 VGCC is primarily involved in their action potential generation in AVP neurons under normotonic conditions.

## Data Availability

The data generated and/or analyzed during the current study are available from the corresponding author on reasonable request.

## Abbreviations

AVP: Arginine vasopressin
VGCC: Voltage-gated Ca^2^^+^ channel
FFA: Flufenamic acid
SON: Supraoptic nucleus
PVN: Paraventricular nucleus
MNCs: Magnocellular neurosecretory cells
OXT: Oxytocin
PACAP: Pituitary adenylate cyclase-activating polypeptide
eGFP: Enhanced green fluorescent protein
GAPDH: Glyceraldehyde-3-phosphate-dehydrogenase
ω-CgTx: ω-Conotoxin GVIA
TTA-P2: 3,5-Dichloro-*N*-[1-(2,2-dimethyl-tetrahydro-pyran-4-ylmethyl)-4-fluoro-piperidin-4-ylmethyl]-benzamide
TTX: Tetrodotoxin
